# SYNTHESIS-Breast: A prospective early-phase trial of a genetic-interaction- focused computational algorithm in advanced metastatic breast cancer

**DOI:** 10.21203/rs.3.rs-9522272/v1

**Published:** 2026-05-26

**Authors:** Aanika B. Warner, Britanny B. Solarz, Miroslava Belyov, Valentina Bolanos, Ann McCoy, Takeo Fujii, Jung-Min Lee, Kevin Conlon, Mireya Gomez, Bernadette Redd, Christopher A. Febres-Aldana, Liqiang Xi, Mark Raffeld, Ruchi Patel, Lisa Cordes, William D. Figg, Gal Dinstag, Ranit Aharonov, Tuvik Beker, Kenneth Aldape, Eytan Ruppin, Stanley Lipkowitz, Padma Sheila Rajagopal

**Affiliations:** National Cancer Institute; National Cancer Institute; National Cancer Institute; National Cancer Institute; National Cancer Institute; National Cancer Institute; National Cancer Institute; National Cancer Institute; National Cancer Institute; National Institutes of Health; National Cancer Institute; National Cancer Institute; National Cancer Institute; National Cancer Institute; National Cancer Institute; National Cancer Institute; Pangea Biomed; Pangea Biomed; Pangea Biomed; National Cancer Institute; National Cancer Institute; National Cancer Institute; National Cancer Institute

## Abstract

SYNTHESIS-Breast is an exploratory trial that adapts early-phase design to identify off-label therapies in metastatic breast cancer via ENLIGHT, a retrospectively validated computational algorithm, and generate preliminary data for future trials. ENLIGHT selects treatments via gene-expression-based synthetic lethality/rescue. SYNTHESIS-Breast’s design includes algorithm-specific adaptations (ex. a reproducible molecular tumor board or layered Simon two-stages for fast interim checkpoints). SYNTHESIS-Breast will not only guide ENLIGHT applications, but also future prospective algorithm trials.

## Introduction

Precision oncology, which tailors therapy by matching patients to treatments using tumor-specific genetics, has transformed certain cancer care contexts but remains limited in advanced metastatic breast cancer (MBC). There are few effective later-line personalized options for patients with advanced metastatic triple negative breast cancer (TNBC) or endocrine-resistant hormone-receptor positive (HR+) disease (1). Precision oncology in breast cancer typically relies on targeting tumors with specific DNA alterations, such as germline *BRCA 1/2* variants (2) or *PIK3CA* mutations (3). Tumor-agnostic precision biomarkers, such as high microsatellite instability (MSI-H) (Vidula 2022), high tumor mutational burden (TMB-H) (4), or *NTRK/RET* (5,6) gene fusions, are uncommon in advanced metastatic breast cancer, leaving most patients with chemotherapeutic options of limited efficacy (7).

The historic one-alteration, one-treatment paradigm limits eligibility in advanced metastatic cancers. The NCI-MATCH trial used DNA-based alterations to match patients with advanced cancers to targeted therapies. Of the total population, 37.6% had actionable mutations and 17.8% were matched to treatment. (8) The breast cancer-specific cohort match rate was 13.7%. Other precision oncology trial efforts, such as I-PREDICT (9) and Personalized OncoGenomics (10) also reported limited breast cancer-specific match rates (5% and 25.6%, respectively). These results highlight the comparable gatekeeping of eligibility for treatment options for advanced breast cancer through the classical precision strategy.

Even when studies of targeted therapies are negative overall, specific patients not selected by current biomarker strategies demonstrate responses. For example, while the phase II FERGI trial in HR+ MBC evaluating the PI3K inhibitor pictilisib did not demonstrate improvement in progression-free survival (PFS), approximately 25% of patients in the treatment arm experienced clinical benefit compared with placebo (11). Similarly, the phase III KEYNOTE-119 trial evaluating pembrolizumab in later line advanced metastatic TNBC did not show an overall survival benefit, but did show improved efficacy among patients with persistently PD-L1 enriched tumors (13). These results reinforce the ongoing need for better strategies to identify responders, an issue well known for immunotherapy.

Response rates in advanced cancers, even those treated using precision therapy strategies, are also not as high as often anticipated by providers or patients(14). Targeted therapies do supersede overall response rates and survival rates compared to chemotherapies (15). Absolute responses to these drugs, however, typically range from 35–60% and decrease with subsequent lines of therapy (16). These issues point to the need for strategies that can expand upon the one-alteration, one-treatment framework for a more robust response.

Strategies that integrate multiple data types are routinely explored in computational oncology but rarely make it to the clinic. One key barrier is the lack of approaches to prospectively assess computational algorithms. Trials – even with novel designs – classically center on one biomarker, one treatment and associated response. One potential approach that expands the one-alteration, one-treatment framework to consider the genetic interactions of drug target genes via synthetic lethality (SL). This is a paradigm wherein concurrent disruption of two genes causes cell death, while each individual alteration is tolerated (17). This mechanism underlies the success of PARP inhibitor therapy, which is already used in breast cancer(18).

Gene expression-based interactions predict treatment response and resistance (19). The ENLIGHT (Expression Networks for highLIGHting Tumor vulnerabilities; Pangea Biomed) algorithm uses large-scale SL *in vitro* and *in vivo* data, combined with individual-level RNA sequencing, to predict response to a given targeted therapy or immunotherapy based on anticipated induction of SL (20). Unlike DNA-only approaches, ENLIGHT captures functional activity through the whole transcriptome and predicts both sensitivity and resistance to drugs, offering a completely different potential mechanism to identify therapeutic options for patients. This algorithm has been retrospectively validated for predicting treatment responders using data from 21 trials (20). The positive predictive value of ENLIGHT match in predicting response to a given therapy in breast cancer ranged from 40–71%. However, to date, there is no way to prospectively evaluate this, or comparable, algorithms in an adequate timeframe relevant for future potential clinical deployment.

This paper describes SYNTHESIS-Breast, an exploratory early-phase feasibility study that prospectively identifies potential off-label treatment options for participants with heavily pretreated metastatic TNBC and endocrine-refractory HR+ breast cancer. SYNTHESIS-Breast provides a systematic, prospective evaluation of ENLIGHT and SL via gene expression as a treatment selection strategy. More broadly, this design also offers a potential model for conducting early-phase precision oncology trials of computational algorithms in a reasonable timeframe.

## Methods and Unique Trial Elements

### Study Design, Objectives, and Participant Eligibility Criteria

SYNTHESIS-Breast (NCT07067138) is a prospective, non-randomized, early-phase pilot trial designed to evaluate feasibility and clinical performance of the ENLIGHT algorithm for treatment selection in advanced MBC. Participants may be matched to one of 22 off-label treatment options with FDA approvals in other indications. These 22 therapies were selected based on their availability at the NIH Clinical Center. The primary objective of the study has two parts: (1) to assess the feasibility of using the ENLIGHT algorithm to match heavily pretreated participants with advanced MBC to treatment options and, (2) if feasible, to assess the objective response rate (ORR) among those treated with any option selected using the ENLIGHT algorithm. Importantly, the algorithm itself is treated as the investigational intervention rather than the therapies. This trial is not intended to support ENLIGHT's immediate clinical deployment, but rather to gather preliminary data for future clinical contexts in which ENLIGHT could be deployed.

**Figure 1:**
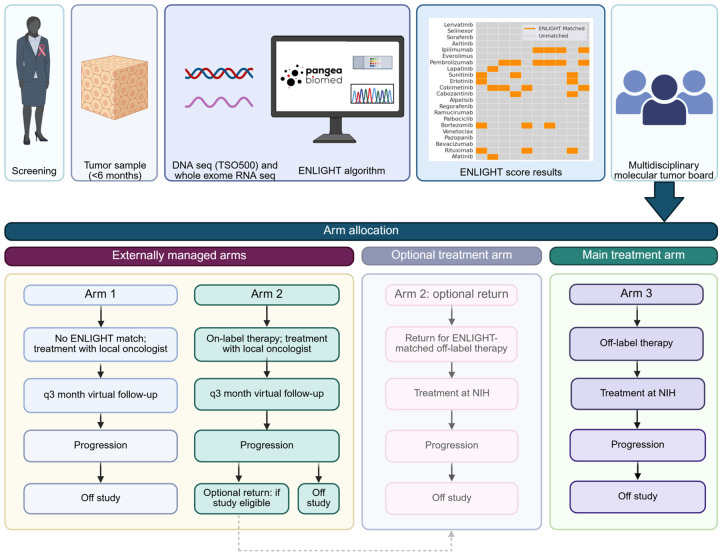
Trial schema of SYNTHESIS-Breast. A participant is screened when tumor biopsy samples undergo genomic/transcriptomic profiling via the **TruSight Oncology 500 (TSO500)** platform. Transcriptomic data is analyzed by **ENLIGHT** (Pangea Biomed). The participant is reviewed by multidisciplinary molecular tumor board (MTB) and assigned to one of 3 arms.
**(External) Arm 1**: No treatment recommendation → treatment per local oncologist and followed virtually**(External) Arm 2**: Recommendation for FDA on-label treatment option → treatment per local oncologist and followed virtually. If progression occurs, participants have the potential option for ENLIGHT-matched therapy if they still meet screening criteria.**(Treatment) Arm 3**: ENLIGHT recommendation of 1 of 22 off-label treatments. Participants are treated and followed at the NIH Clinical Center with imaging every 2 cycles until progression / other off-study event.

Study design is shown in Figure 1. There are two cohorts: TNBC and endocrine-refractory HR+/HER2−. Within each cohort are three possible arms:

Main treatment arm:
  - **Transcriptomic match (Arm 3)**: There are no FDA-approved on-label options available, and there is at least one ENLIGHT-matched treatment option available. Participants are treated at the NCI.

Externally managed arms:
  - **No match (Arm 1)**: No FDA-approved on-label options or ENLIGHT-recommended therapy available. Participants are treated with the treatment as chosen by their home oncologist and followed by virtually the research team.
  - **Genomic match (Arm 2):** FDA-approved on-label targeted therapy available based on standard genomic markers. Participants are treated in collaboration with their home oncologist and followed with the research team virtually. Participants in Arm 2 who have a genomic match may be reconsidered for an ENLIGHT-matched therapy after progression on the genomically selected treatment if they continue to meet eligibility criteria (Arm 2, optional return).

Participants in the external arms (Arms 1 and 2) are followed remotely every three months until progression or for up to two years, while participants in the main treatment arm (Arm 3) are followed remotely for up to two years after progression on study treatment. Study arms are not compared directly. All are intended to gather information about the best potential uses of ENLIGHT.

Eligible participants include adults (≥18 years) with histologically confirmed, measurable TNBC or endocrine-refractory HR+/HER2− advanced metastatic breast cancer. Participants must have been treated with at least one line of systemic therapy after diagnosis of metastatic disease, anticipate or have progressive disease or adverse events requiring discontinuation of their current regimen, and must not be able to transition to another approved systemic therapy shown to improve overall survival. Archival tumor must be available within the past 6 months, or participants must be amenable to undergo tissue biopsy (which can be completed while on therapy). This constraint on biopsy timing is required to optimize reliability of the ENLIGHT results while being pragmatic about repeat biopsies in these patient populations. Participants must have ECOG 0–2, stable organ function without crisis, controlled comorbid illnesses, and meet expanded lab cutoffs. Full inclusion and exclusion criteria are provided in the **Supplementary Appendix.**

This clinical trial is being performed in accordance with the Declaration of Helsinki, U.S. Common Rule (45 CFR 46) and after approval by the NIH IRB and in accordance with an assurance filed with and approved by the U.S. Department of Health and Human Services. This study’s IRB number is IRB001891; the trial was approved 5/2025. informed written consent is being obtained from each participant or each participant's representative.

### Pragmatic Molecular Profiling and ENLIGHT

For the ease of participants and referring providers, biopsy samples may be mailed. Received tumor samples will be processed through the CLIA-certified NCI Laboratory of Pathology and undergo sequencing on the TruSight Oncology 500 (TSO500) platform, which includes a 523-gene tumor DNA panel and whole-exome RNA-seq panel. De-identified RNA sequencing data is securely transferred to Pangea Biomed to run the ENLIGHT algorithm.

The ENLIGHT algorithm generates ENLIGHT Matching Scores that are used to prioritize each of the 22 targeted or immune therapies available through the study. An example of the report output is provided in the **Supplementary Appendix.**

### Molecular Tumor Board and Arm Assignment with Reproducible Criteria

A multidisciplinary molecular tumor board (MTB) reviews each case with clinical and histopathologic data, sequencing data, and ENLIGHT report information before finalizing treatment recommendations. The MTB includes the principal investigator with at least one other NIH-based medical oncologist, pharmacist, molecular pathologist, and research nurse.

For reproducibility, the MTB must explicitly review the following elements as per the protocol:

- ENLIGHT predicted match strength for a given therapy
- Best evidence available based on existing approvals in other indications and safety data in breast cancer
- Known comorbidities, drug-drug interactions with participant medications, and treatment history
- Potential treatment toxicities
- Availability of therapy in a timely fashion

If more than two treatments receive a similar ENLIGHT score, the MTB will use an explicitly scored charter to prioritize up to two options (Table 1).

From tissue receipt at the Laboratory of Pathology through ENLIGHT analysis and MTB review, the total turnaround time is tracked to be less than 30 days to facilitate participants’ transitions to next line of therapy without extensive delays.

There are two initial visits: a screening/consent visit and a reporting visit that occurs after the MTB. At the reporting visit, participants learn their arm assignment and, if in Arm 3, discuss the ENLIGHT-matched treatment option. To facilitate broad participation, screening/consent and reporting visits are virtual, so that in-person visits are limited to those in Arm 3 undergoing active treatment at the NCI.

### Endpoints, Sample Size, and Other Statistical Considerations

**Figure 2:**
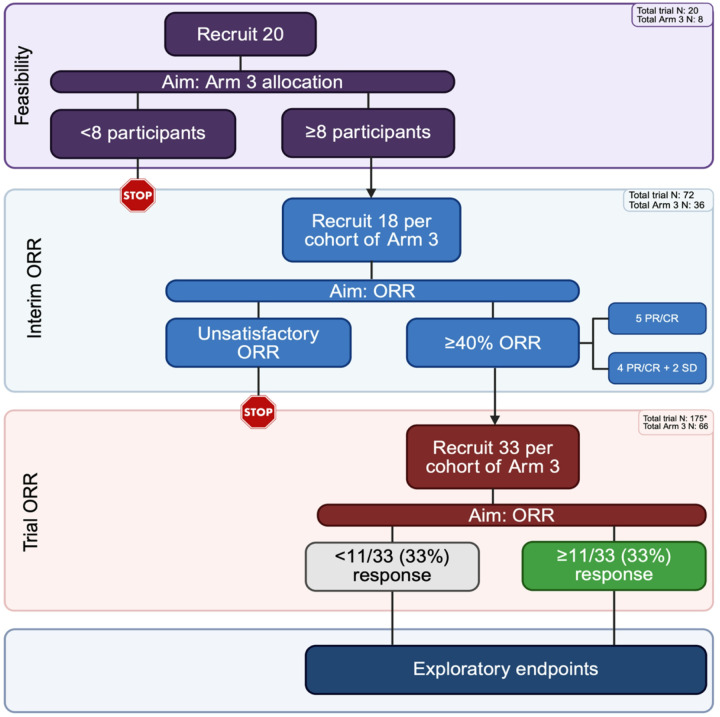
Trial Objectives and Endpoints with Nested Simon Minimax two-stage Design. The trial primary objective is two parts: (A) feasibility of matching participants to an ENLIGHT-directed therapy, and (B) objective response rate (ORR) to a therapy selected with support from ENLIGHT. **Objective part 1 - Feasibility:** After the first 20 successfully screened participants, if ≥8 are assigned to Arm 3, the study proceeds. **Objective part 2 – ORR:** Each cohort (TNBC, HR+/HER2–) enrolls 18 participants initially including those included in part 1; if ≥5 respond, accrual continues to 33 per cohort. The trial is successful per cohort if ≥11/33 (33%) respond. **Total screened:** Approximately 175 participants will be screened (estimated 50% Arm 3, 30% Arm 2, 20% Arm). Anticipate feasibility/primary objective can be completed within 1 year and total trial within 4 years based on average clinic volume. Secondary/exploratory objectives are descriptive and intention-to-treat; ORR will include 95% CIs.

Per the two-part objective described above, the primary endpoint comprises two parts: (A) A feasibility success threshold based on the proportion of participants assigned to Arm 3, and (B) if feasible, the ORR among Arm 3 participants (Figure 2).

Primary endpoint parts A and B use a nested modified Simon Minimax two-stage design for each cohort (TNBC and HR+ MBC). For part A, arm assignment will be assessed after the reporting visit of the 20th successfully screened participant. If at least 8 participants are assigned to Arm 3, the study will advance to part B.

Part B estimates a null ORR based on the control arms of multiple advanced MBC clinical trials of approximately 20%. Part B has 80% power to detect an improved response rate of 40% or greater. In another modified Simon Minimax two-stage design with α=0.05 and β=0.20, 18 patients will be enrolled per cohort (those from the feasibility lead-in are included) for the first stage. If ≥5 respond, accrual continues to 33 patients per cohort. The trial will be considered successful if ≥11 responses (33%) are observed. Interim analyses will be performed when each cohort has an enrollment of 18 participants in Arm 3, and each participant has completed 2 cycles of therapy. The interim stages and analyses facilitate sufficiently timely checkpoints more appropriate to the timescale of computational algorithms.

We anticipate screening 175 participants based on preliminary estimations of ENLIGHT matching at 50% to the main treatment arm using publicly available data in MBC. Over the course of one year, we estimate 40–60 participants to be accrued (allowing the feasibility lead-in to be completed in a reasonable timeframe for a computational algorithm go/no go) and trial completion with full enrollment within 4 years if all interim stages are passed.

Clinically focused secondary endpoints will focus on match rates of specific treatments, clinical benefit rates, and adverse events. Exploratory endpoints include progression free survival (PFS), overall survival (OS), patient-reported outcomes (PROs), and correlations with clinical molecular measures and experimental molecular measures (including spatial transcriptomics and immune subset features). Secondary and exploratory endpoints will be descriptive. Adverse events will be graded by CTCAE v5.0. Biospecimens on study will include serial blood draws for correlation to circulating tumor DNA, immune profiling, and soluble factor analysis, as well as an optional on-treatment biopsy. Detailed objectives, endpoints, and biospecimens being collected on the study are described in the **Supplementary Appendix.**

## Discussion

SYNTHESIS-Breast adapts early-phase drug trial design to prospectively test computational algorithms and uses ENLIGHT, a transcriptome-based algorithm, as a proof of concept.

SYNTHESIS-Breast builds on prior precision oncology efforts, such as NCI-MATCH, I-PREDICT, or the WINTHER RNA-seq-based study in terms of biological approach using the ENLIGHT algorithm (8,9,21). These prior studies relied on individual matches to specific genomic alterations. ENLIGHT predicts treatment response using multiple genetic interactions (20). By anchoring predictions in genetic interactions across the transcriptome, ENLIGHT leverages potential vulnerabilities that are not evident through mutation-based analyses alone (22).

SYNTHESIS-Breast also introduces unique trial design elements given its focus on computational algorithms rather than drugs. Clinical decision support technology is often justified for use via retrospective/observational evidence (as with many FDA-approved artificial intelligence software tools) or large phase 3 clinical trials requiring enormous resources (as was used to approve Oncotype in breast cancer care) (23,24). Retrospective/observational evidence may fail to account for deployment feasibility, applicability across various clinical settings and populations, operational constraints, or evolving clinical landscapes (25). Phase 3 clinical trials, besides their resource requirements, take years to read out and are not practical for fast-moving computational algorithms. There is no clear framework for a flexible, time-limited, go/no-go strategy to generate preliminary data to determine if a computational algorithm is worth advancing. Our use of nested Simon 2-stage designs, including feasibility as a part of the primary objective, creates multiple checkpoints that can be assessed relatively quickly over the course of the study. Our molecular tumor board has a quantitative, reproducible scoring system in the event of multiple possible therapeutic options. Our focus on the algorithm, as opposed to the drugs-responses, does limit the statistical conclusions we can draw from this study, but also allows us to observe predicted responses across 22 targeted therapies or immunotherapies and generate comparatively quick initial data for future, more traditional studies.

SYNTHESIS-Breast importantly also features multiple patient-centered and pragmatic design elements. Virtual visits minimize patient burden. Archival biopsy samples from the past 6 months seek to compromise between gene expression data being more accurate closer to time of collection and pragmatic accommodation to avoid multiple recurrent tissue procedures in close proximity. We allow for continued therapy while testing is ongoing for patient comfort and anxiety. We intentionally follow up on patients who do not match and collect data to learn more about who is not served by this approach for future work.

DNA-centric, mutation-centered strategies improve outcomes for select subsets but often insufficiently address the challenges of advanced disease, particularly in advanced metastatic breast cancer. SYNTHESIS-Breast (NCT07067138) is a prospective, non-randomized, early-phase pilot trial designed to evaluate feasibility and clinical performance of the ENLIGHT algorithm for treatment selection in advanced MBC. ENLIGHT may provide complementary predictive information that simply does not otherwise exist with current predictive biomarkers. If successful, we hope that this trial supports the advancement of transcriptomic data in the clinic, as well as computational algorithms in prospective clinical trials.

## Supplementary Material

Supplementary Files

This is a list of supplementary files associated with this preprint. Click to download.

- 20260421SYNTHESISNPJSupp.docx

## Figures and Tables

**Table 1:** Quantitative Criteria for Molecular Tumor Board with > 2 comparable ENLIGHT matches

Category	Criteria	Scoring
ENLIGHT match	ENLIGHT match strength	Match: 0 Strong Match (>80%): +1
Evidence based in breast cancer	Level of existing breast cancer data demonstrating any responders (acceptable if overall negative study)	Tested up to Phase 1: +1 Tested up to Phase 2: +2 Tested up to Phase 3: +3
Prior use in breast cancer	Whether therapy has been used in any breast cancer context	No: 0 Yes: +1
Availability at NIH pharmacy	Drug availability and procurement requirements	Currently available: 0 Needs ordering or shortage: −1
Comorbidities and contraindications	Presence of medical conditions that may affect tolerability	No: 0 Yes: −1 for each condition or contraindication
Drug-drug interactions	Known or potential interactions requiring modifications or discontinuation	No: 0 Yes: −1 for each medication with concern
Prior exposure to recommended therapy	Previous treatment with same drug or within same drug class	No: 0 Yes: −1 if <2 cycles of response or treatment within the past 6 months

## Data Availability

Data is currently being generated by the authors and is not publicly available due to patient privacy restrictions in the setting of an ongoing clinical trial, but can be discussed with the corresponding authors. De-identified participant data will be made available at the time of study completion through public repository in accordance with the NIH Data Management and Sharing Policy.

## Code Availability

ENLIGHT has been previously published (Dinstag et al. *Med* 2023). A public interface to use ENLIGHT is available at https://ems.pangeabiomed.com/.
